# Recent Advancements in In Vitro Fertilisation

**DOI:** 10.7759/cureus.30116

**Published:** 2022-10-10

**Authors:** Kadambari Singh, Deepika Dewani

**Affiliations:** 1 Surgery, Jawaharlal Nehru Medical College, Datta Meghe Institute of Medical Sciences, Wardha, IND; 2 Obstetrics and Gynaecology, Jawaharlal Nehru Medical College, Datta Meghe Institute of Medical Sciences, Wardha, IND

**Keywords:** in vitro fertilization (ivf), reproductive genetics, pre-implantation genetic screening, letrozole, co-enzyme q10, infertility

## Abstract

The field of assisted reproductive technologies has witnessed many new developments over the past 10 years. This review examines new stimulation techniques that might increase the number of fully developed oocytes derived during the in vitro fertilisation (IVF) cycle in addition to strategies for enhancing oocyte quality in older women.

Before moving on to several fresh methods for determining endometrial receptivity, we talk about how preimplantation genetic screening (PGS) is currently being utilised. The main goal of this review is to highlight technological fields that might be debatable or are still sufficiently novel to require rigorous controlled trials for recognition. The use of IVF has been on the rise recently, mostly as a result of deferred childbearing, and there is no reason to believe that this trend will alter. Infertility therapies have advanced significantly thanks to the methods and techniques that were established via studies on animals and, more recently, people. Some technical discoveries in reproductive medicine have had a significant impact on innovations and treatment choices in other fields of medicine as well. The objective of this succinct review article is to quickly summarise and explain the advancements made in this intriguing area of medicine over the past 40 years.

## Introduction and background

A higher yield of mature oocytes during an in vitro fertilisation (IVF) cycle may be obtained by using novel stimulation techniques. Additionally, we are interested in devising techniques to raise oocyte quality, particularly in older women. In fact, according to recent projections, assisted reproductive technologies (ART) may keep 400 million people (3% of the world's population) alive by the year 2100 [1]. Treatments must be both secure and efficient as a result. IVF research will face new challenges in the future. The main difficulties are as follows: how to deal with the inevitable issues of egg ageing and female infertility, how to understand implantation problems and subsequently create remedies, and how to advance therapies for male infertility. Given the compelling motivations for caretakers, researchers, and most critically, infertile couples, only time will tell what chances and avenues the ensuing 40 years will offer for assisted reproduction. Since the world's first IVF baby was born around 40 years ago, it is estimated that over eight million infants have been born as a result of IVF infertility therapy. Before delving into some problematic new techniques for determining endometrial receptivity, we first explore the current debate around preimplantation genetic screening (PGS) [1]. Due to sociodemographic shifts and advances in technology, the demand for IVF has increased, changing how a significant portion of the population reproduces. The goal of this analysis is to emphasise the social and demographic factors that are fueling an increase in the demand for IVF on a global scale, in addition to providing an overview of emerging technologies that have the potential to considerably enhance IVF usage and lower its cost.

## Review

Improving oocyte quality: role of mitochondria

**Figure 1:**
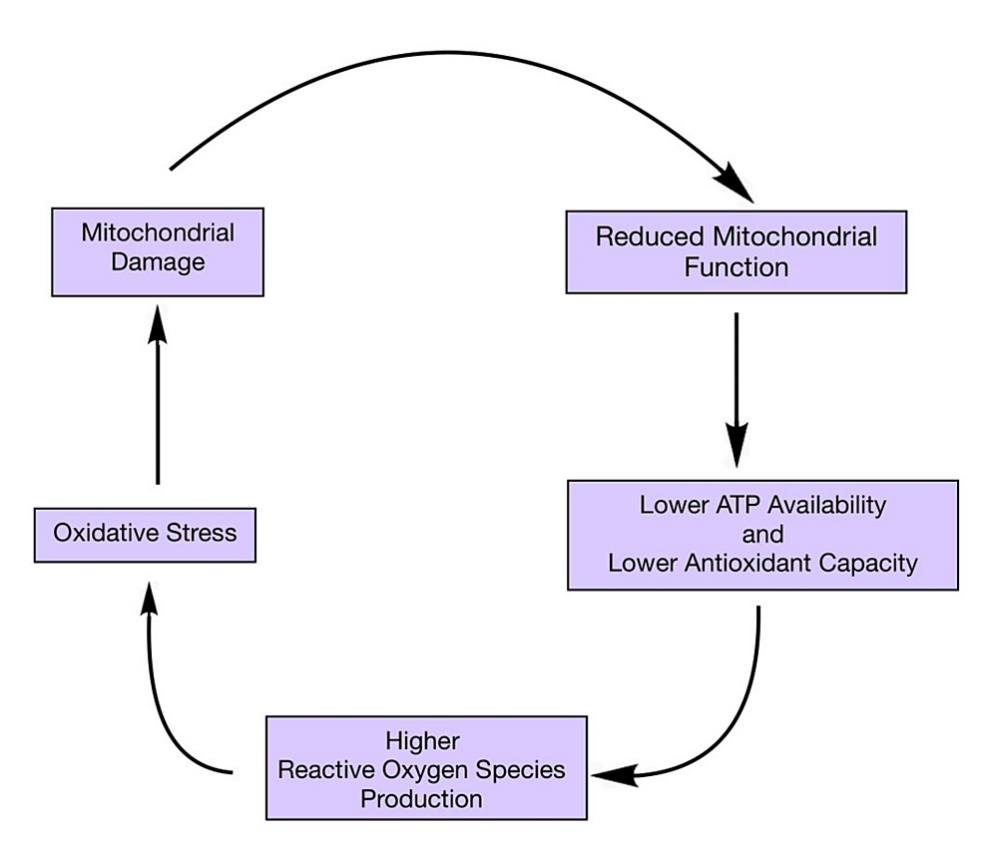
Virulent cycle between mitochondria dysfunction and oxidative stress damage Mitochondria play an integral role in maintaining oocyte quality [6]

A woman's procreative capacity dramatically declines in the fourth decade of her life, which is directly tied to an ageing-related decline in oocyte quality and quantity [2]. After the age of 32, fecundity gradually decreases, and after the age of 38, it decreases fast [3]. Since the frequency of live births following oocyte donation in older women is proportional to the donor's age, oocyte quality is most likely the key factor causing a decrease in fecundity with age. Although greater DNA damage brought on by a less active DNA repair mechanism is a potential contributor to oocyte loss, the pathways leading to an increase in ovarian follicle loss in "aged" ovaries are yet unknown [4]. Chromosome aneuploidy increases in frequency when oocyte quality declines, primarily as a result of meiotic mistakes made during oocyte maturation. The oocyte must undergo nuclear, cytoplasmic, and epigenetic alterations in order to develop. These modifications all need energy, which the mitochondria provide by oxidative phosphorylation (OXPHOS) [5]. Mitochondria play an integral role in maintaining the quality of oocytes, the dysfunction of which is depicted in the flow chart given below [6] (Figure 1).

Coenzyme Q10 supplementation

Adenosine triphosphate (ATP) is made through an approach called OXPHOS, which involves five complexes that are positioned on the inner mitochondrial membrane [2].

**Figure 2:**
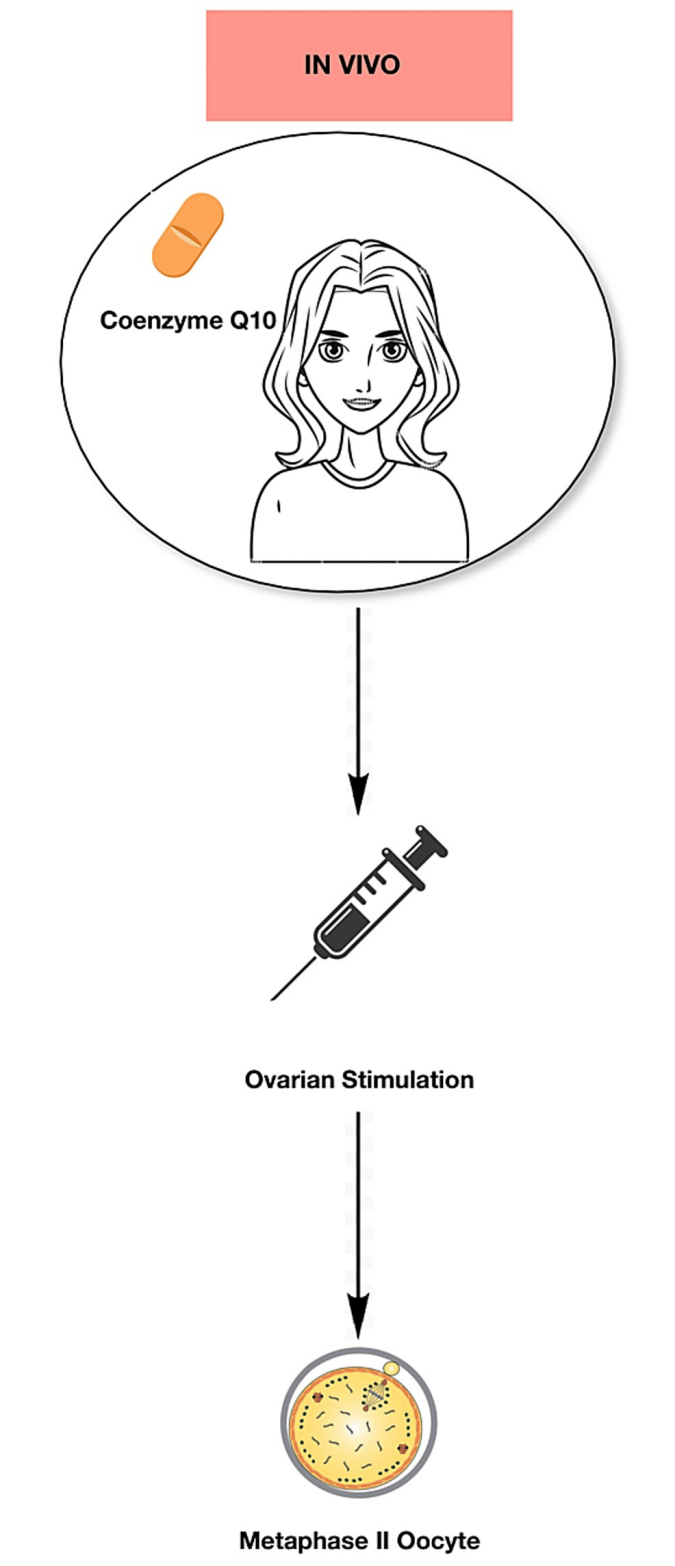
In vivo method of CoQ10 supplementation CoQ10 is directly administered to the patient in the form of oral tablets [6] CoQ10: coenzyme Q10

**Figure 3:**
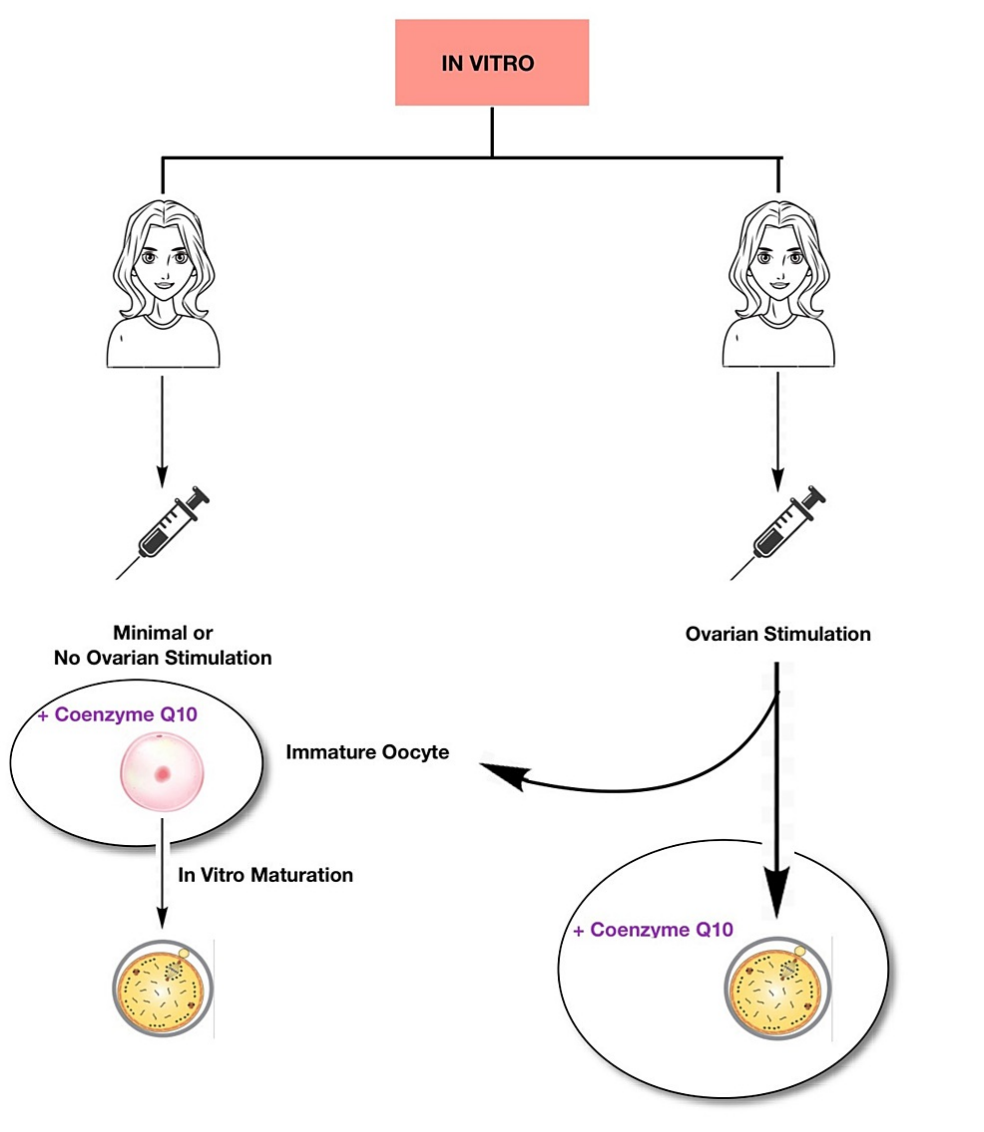
In vitro method of CoQ10 supplementation CoQ10 is supplied to the culture media containing oocytes [6] CoQ10: coenzyme Q10

The antioxidant properties of ubiquinone, also known as coenzyme Q10 (CoQ10), along with its capacity to control cellular redox and having an impact on several signalling pathways make it crucial in this process [7,8]. After the age of 30 in humans, the majority of their tissues have lower CoQ10 concentrations [9,10]. Due to its association with a decline in fertility and an increase in aneuploidies, CoQ10 loss may hasten the ageing process. In an elderly animal model, Ben-Meir et al. (2015) showed that supplementing with CoQ10 prevented the loss of ovarian reserve, enhanced mitochondrial function, and markedly decreased oocyte aneuploidy. In comparison to older animals on a placebo, these elderly mice stimulated produced more offspring and had more oocytes [2]. Afterwards, it was found that isolated CoQ deficiency brought on by conditional deletion of the PDSS-2 gene in young animal oocytes resulted in phenotypic changes resembling oocyte mitochondrial dysfunction linked to ageing [2]. If the animals received CoQ10, these alterations might be undone. It is reasonable to assume that CoQ10 supplementation would be beneficial to older women in a manner similar to how CoQ10 administration has been shown to improve reproductive outcomes in elderly animals [2]. Currently, a great deal of research is being conducted in this field. The ageing process is very different between mice and women because of the enormous differences in lifespan, despite the animal model's apparent promise. Given that giving CoQ10 to mice for 12-16 weeks is likely equivalent to years of human use, more clinical research is necessary [2]. Ultimately, we can say that CoQ10 is advantageous in IVF as it improves the oocyte quality with regard to ageing. The only disadvantages of this method are the side effects of CoQ10, which are very rare and include heartburn, nausea, diarrhoea, abdominal pain, fatigue, and dizziness. This procedure of supplementing CoQ10 can be accomplished by two methods: in vivo (Figure 2) and in vitro (Figure 3). In in vivo method, oral treatment methods are employed to enhance the oocyte quality. Whereas, in in vitro method, the culture media is supplemented with the enzyme, which can further be either standard culture or in vitro maturation culture [6].

Mitochondrial transfer

To combat ooplasmic ageing, subcellular oocyte modification has been used in other programmes. Cohen et al. performed ooplasmic transfers into mature oocytes of patients whose numerous IVF cycles had failed due to insufficient embryo development using donor oocytes. The findings indicated that the infants were alive and in good health [11]. Given that mitochondria are found in the cytoplasm, donor ooplasm's mitochondria were also present in recipient eggs, which was thought to be the primary factor influencing better development. Ooplasm donation is no longer practised because heteroplasmy testing on several healthy children revealed that their mitochondrial DNA (mtDNA) was derived from both the mother and the cytoplasm donor, indicating that the heteroplasmy was present in the oocytes [12]. But later research using autologous mitochondrial transfer has enhanced the earlier work with ooplasm transfer. The mitochondria are taken out of the patient's ovary's oocyte precursor cells in the superficial epithelial layer and injected into their oocytes during fertilisation in this cutting-edge procedure. It has been demonstrated that mitochondrial injection enhances embryo growth and aids in live births in women who had previously experienced poor embryo development [13]. There are some unethical demerits regarding the above-mentioned technique, such as germlines being modified during mitochondrial donation which leads to the passing down of such modifications to upcoming generations. This method also has the potential of psychological and emotional impact on the offspring leading to an effect on an individual's sense of identity. The efficacy of this method, like the existence of oocyte precursor cells, needs to be confirmed through appropriate randomised controlled trials.

Medical advancements

To enhance the production of oocytes available for IVF, controlled ovarian hyperstimulation, or COH is used. Numerous gonadotropin injections, visits to the fertility clinic, and transvaginal ultrasound examinations are required for COH. As a result, it takes a lot of time and effort to perform COH. The utilisation of transportable facilities and perhaps self-contained endovaginal telemonitoring might further simplify follicular and endometrial monitoring in light of recent improvements in portable, less expensive ultrasound systems [14]. Combining such methods would hasten and reduce COH intrusion. This method of treatment is known to have several demerits such as emotional stress, high economical costs, and lastly ovarian hyperstimulation syndrome (OHSS), which manifests as abdominal pain, nausea, vomiting, bloating, tenderness over the area of ovaries, diarrhea, and shortness of breath. Evaluation of patients for psychological issues during IVF is one therapy that may help lessen the burden of treatment even further, in addition to counselling and coping mechanisms like e-therapy [15,16]. As per a randomised controlled trial conducted by van Dongen et al., internet-based interventions carry great potential in relieving psychological distress, particularly when care is personalised to patients' personal risk profiles [17].

Technological advancements

One could argue that automation and the miniaturisation of IVF laboratories are the two most promising technological advancements that have the potential to democratise access to IVF in the near future. An IVF facility's exorbitant costs, unequal access, and inconsistent operations are primarily the result of its manual hiring, construction, and operation.

The fundamental steps conducted in the IVF laboratory are as follows. Firstly, there is determination and segregation of sperm and oocytes, followed by fertilisation and embryo culture. Next, we select embryo for transfer, and lastly, cryopreservation of surplus embryos and gametes is done.

Significant progress has already been made toward automating each of these various stages with the help of novel techniques. However, the majority of the IVF process is still carried out manually. The revolutionary new IVF lab-on-a-chip concept has the potential to transform in vitro fertilisation by automating nearly all necessary steps in a single system [15,16,17]. In the multidisciplinary field of microfluidics, fluid dynamics is precisely controlled and manipulated under the influence of minute geometrical constraints that favour surface forces over volumetric counterparts. Earlier IVF laboratory procedures used macroscale methodologies to microscale cellular biological activities, despite their historical success [18].

At least four benefits that could result from integrating microfluidics into the IVF laboratory have been predicted. Firstly, fluidic gamete/embryo manipulations that are precisely controlled. The second involves creating biomimetic culture environments, while the third entails making microscale genetic and molecular bioassays easier. The last benefit involves allowing for miniaturisation and automation. On the contrary, it is difficult to standardise and scale up, which require external pumps and tubing, as well as connectors and valves to operate. Automated sperm analysers and microfluidic sperm-sorting equipment are frequently used in IVF procedures [19,20,21]. Using microfluidics, sperm and sperm-bearing tissue have been removed from testicular biopsies [22,23,24,25,26,27,28]. Even though the vast majority of IVF patients are candidates for conventional fertilisation, microfluidic in vitro insemination has been shown to be successful [29]. Potentially, intracytoplasmic sperm injection (ICSI) will not be necessary in the future thanks to microfluidic devices. ICSI has established itself as the de facto technique of insemination in human clinical IVF, demonstrating the significance of precise microfluidic push/pull cumulus-oocyte-complex cumulus cell removal in creating good visibility of the oocyte cytoplasm/orientation [30]. Despite its technological difficulties, the ICSI phase of fertilisation may be carried out effectively on a commercial scale [30]. In the future, automated ICSI is likely to be integrated with microfluidics, robotics, and high-tech optics [31,32].

Scientific advancements 

Our understanding of the mechanisms that control folliculogenesis has continually improved as a result of research on fertility preservation [33]. The interactions between the oocytes and the somatic cells surrounding them, as well as the vital hormones and growth factors, have been revealed by follicular in vitro culture techniques. Recent developments in multi-step culture techniques have made it possible to activate, develop, and in vitro mature (IVM) ovarian cortex tissue primordia to produce metaphase II oocytes [34]. The advantages of IVM include reduced risk of OHSS and polycystic ovaries, lower medication costs, reduced stress, and lower monitoring burden. In contrast, it has been observed that chances of live birth with IVM are slightly lower than with IVF. Ovarian tissue cryopreservation and IVF have improved the chances of preserving fertility in prepubescent girls and adolescent women who are more likely to experience primary ovarian insufficiency (POI) from gonadotoxic chemotherapy for cancer or other serious illnesses. As long as some dormant follicles are still present in the ovarian cortex, fascinating developments in this technology may make it possible to isolate oocytes from females who have undergone POI or who have gone through natural menopause. An artificial ovary could be developed in a mouse model using scaffolds made through 3D printing for tissue engineering [35,36]. Microfluidic culture techniques can be used to mimic the menstrual cycle by promoting follicle development [37].

Co-treatment with gonadotropins and letrozole in IVF

Gonadotropin stimulation and the oral medication letrozole used during IVF cycles may be beneficial, especially for breast cancer women receiving fertility preservation treatment, according to recent research [38-41]. Letrozole and ovarian stimulation are employed in the treatment of breast cancer patients to reduce blood oestrogen levels. According to these studies, breast cancer patients who received letrozole and gonadotropins for the duration of the stimulation had lower estradiol concentrations than they would have anticipated but also had more mature oocytes available for cryopreservation than breast cancer-free controls who received conventional COH [41]. It has been associated with positive effects, including decreased gonadotropin doses that minimise the cost of IVF therapy and enhanced oocyte and mature oocyte counts while retaining the same pregnancy rate as conventional stimulation. On the other hand, gonadotropin stimulation may lead to OHSS, profound hypoestrogenemia, as well as more time-consuming and complex stimulation protocols. In 2005, the impact of letrozole on intraovarian testosterone levels and the success of IVF cycles was investigated. According to Garcia-Velasco et al., the use of letrozole 2.5 mg during the initial five days of gonadotropin stimulation significantly raised the levels of androstenedione and testosterone in follicular fluid and improved the success of IVF cycles. Letrozole considerably outperformed the control group in terms of both the number of recovered oocytes and the implantation rate.

Pre-treatment with dehydroepiandrosterone/testosterone

Numerous strategies have been investigated in an effort to raise intrafollicular androgen concentrations in people who do not respond well to medication because intraovarian androgens may significantly impact early follicular development. To increase the ovarian sensitivity to FSH and follicular response to gonadotrophin therapy in low-responder IVF patients, transdermal testosterone was utilised as a pre-treatment [42]. As the quantity of cumulus oocyte complexes grew, so did the frequency of clinical pregnancies and live deliveries. According to Gleicher et al., individuals with low ovarian reserve received dehydroepiandrosterone (DHEA) for 30 to 120 days as a supplement (25 mg three times per day). They found that individuals who had received treatment had higher anti-mullerian hormone (AMH) levels and greater conception rates when compared to patients who had not received treatment. DHEA may lessen aneuploidy and miscarriage, according to the same study team's hypothesis [43,44]. Wiser et al. carried out a prospective randomised controlled experiment to ascertain the effect of DHEA supplementation on the effectiveness of IVF in patients who had poor responses. They discovered that the DHEA group had much higher rates of live birth and higher-quality embryos compared to the controls. In both groups, the number of zygotes and eggs was the same. It is unknown whether DHEA helps older women or persons who respond slowly because not many randomised controlled studies have been conducted on these populations.

New approaches to assess endometrial receptivity

The endometrial receptivity array (ERA), a microarray study of implantation-associated gene expression, and ultrasound assessment of sub-endometrial wave frequency are novel techniques for evaluating endometrial receptivity in IVF facilities.

Reproductive genetics

IVF and reproductive genetics are long considered the industry's frontiers,. Preimplantation genetic testing (PGT) of embryos to find chromosomal abnormalities has become more popular as a result of the introduction of next-generation sequencing. The use of PGT for monogenic disorders has expanded along with the popularity of infertile couple carrier screening. It has many advantages such as improved embryo selection, preventing transfer of embryos that will not implant, less time-consuming procedures, reduced costs, and lastly, it has a positive impact on psychological well-being. However, being an invasive procedure is one of its major disadvantages. Other demerits include a cycle with no transfer and embryo mosaicism. Future treatments for severe monogenic disorders may employ germline genome modification (GGM), thanks to advancements in micromanipulation methods and CRISPR-Cas9 gene-editing tools [45]. The UK is currently conducting clinical trials for mitochondrial replacement therapy (MRT), a more advanced treatment than GGM for heritable mtDNA problems [46].

## Conclusions

In the future, more and more people will use IVF, thereby changing the way a large portion of the human population reproduces. In the near future, IVF will likely be used in several regions worldwide to conceive up to 10% of all children. Given the rapid advancement of reproductive genetics and IVG science and technology, it is essential that regulatory organisations and the general public work together to develop a framework for evaluating the moral implications of emerging technologies. Emerging technologies should be incorporated into clinical practice with the help of a carefully planned clinical trial. The IVM technique reduces the risk of OHSS while enhancing patient safety. IVM may also be advantageous for patients who need to preserve their fertility or who do not respond well to other mentioned treatment modalities. It is now evident that technological advancement, the evolution of necessary tools, as well as the accumulation of experience and training among those performing the procedure have all contributed to success rates rising to as much as 56%. For women over the age of 35 years, this technique is feasible.

## References

[1] Reigstad MM, Storeng R (2019). Development of in vitro fertilization, a very important part of human reproductive medicine, in the last 40 years. Int J Womens Health Wellness.

[2] Ben-Meir A, Burstein E, Borrego-Alvarez A (2015). Coenzyme Q10 restores oocyte mitochondrial function and fertility during reproductive aging. Aging Cell.

[3] O'Connor KA, Holman DJ, Wood JW (1998). Declining fecundity and ovarian ageing in natural fertility populations. Maturitas.

[4] Titus S, Li F, Stobezki R (2013). Impairment of BRCA1-related DNA double-strand break repair leads to ovarian aging in mice and humans. Sci Transl Med.

[5] Dumollard R, Ward Z, Carroll J, Duchen MR (2007). Regulation of redox metabolism in the mouse oocyte and embryo. Development.

[6] Rodríguez-Varela C, Labarta E (2021). Does coenzyme Q10 supplementation improve human oocyte quality?. Int J Mol Sci.

[7] Crane FL (2001). Biochemical functions of coenzyme Q10. J Am Coll Nutr.

[8] Quinzii CM, López LC, Gilkerson RW (2010). Reactive oxygen species, oxidative stress, and cell death correlate with level of CoQ10 deficiency. FASEB J.

[9] Morré DM, Guo F, Morré DJ (2003). An aging-related cell surface NADH oxidase (arNOX) generates superoxide and is inhibited by coenzyme Q. Mol Cell Biochem.

[10] Miles MV, Horn PS, Tang PH, Morrison JA, Miles L, DeGrauw T, Pesce AJ (2004). Age-related changes in plasma coenzyme Q10 concentrations and redox state in apparently healthy children and adults. Clin Chim Acta.

[11] Cohen J, Scott R, Alikani M (1998). Ooplasmic transfer in mature human oocytes. Mol Hum Reprod.

[12] Barritt JA, Brenner CA, Malter HE, Cohen J (2001). Mitochondria in human offspring derived from ooplasmic transplantation. Hum Reprod.

[13] Fakih MH, El Shmoury M, Szeptycki J (2015). The AUGMENTSM treatment: physician reported outcomes of the initial global patient experience. JFIV Reprod Med Genet.

[14] Gerris J, Delvigne A, Dhont N (2014). Self-operated endovaginal telemonitoring versus traditional monitoring of ovarian stimulation in assisted reproduction: an RCT. Hum Reprod.

[15] Weng L (2019). IVF-on-a-chip: recent advances in microfluidics technology for in vitro fertilization. SLAS Technol.

[16] Swain JE, Lai D, Takayama S, Smith GD (2013). Thinking big by thinking small: application of microfluidic technology to improve ART. Lab Chip.

[17] van Dongen AJ, Nelen WL, IntHout J, Kremer JA, Verhaak CM (2016). e-Therapy to reduce emotional distress in women undergoing assisted reproductive technology (ART): a feasibility randomized controlled trial. Hum Reprod.

[18] Smith GD, Takayama S (2017). Application of microfluidic technologies to human assisted reproduction. Mol Hum Reprod.

[19] Quinn MM, Jalalian L, Ribeiro S, Ona K, Demirci U, Cedars MI, Rosen MP (2018). Microfluidic sorting selects sperm for clinical use with reduced DNA damage compared to density gradient centrifugation with swim-up in split semen samples. Hum Reprod.

[20] Parrella A, Keating D, Cheung S, Xie P, Stewart JD, Rosenwaks Z, Palermo GD (2019). A treatment approach for couples with disrupted sperm DNA integrity and recurrent ART failure. J Assist Reprod Genet.

[21] Marzano G, Chiriacò MS, Primiceri E (2020). Sperm selection in assisted reproduction: a review of established methods and cutting-edge possibilities. Biotechnol Adv.

[22] Cho BS, Schuster TG, Zhu X, Chang D, Smith GD, Takayama S (2003). Passively driven integrated microfluidic system for separation of motile sperm. Anal Chem.

[23] Schuster TG, Cho B, Keller LM, Takayama S, Smith GD (2003). Isolation of motile spermatozoa from semen samples using microfluidics. Reprod Biomed Online.

[24] Nosrati R, Vollmer M, Eamer L, San Gabriel MC, Zeidan K, Zini A, Sinton D (2014). Rapid selection of sperm with high DNA integrity. Lab Chip.

[25] Wu JK, Chen PC, Lin YN, Wang CW, Pan LC, Tseng FG (2017). High-throughput flowing upstream sperm sorting in a retarding flow field for human semen analysis. Analyst.

[26] Nagata MP, Endo K, Ogata K (2018). Live births from artificial insemination of microfluidic-sorted bovine spermatozoa characterized by trajectories correlated with fertility. Proc Natl Acad Sci U S A.

[27] Mangum CL, Patel DP, Jafek AR (2020). Towards a better testicular sperm extraction: novel sperm sorting technologies for non-motile sperm extracted by microdissection TESE. Transl Androl Urol.

[28] Samuel R, Feng H, Jafek A, Despain D, Jenkins T, Gale B (2018). Microfluidic-based sperm sorting & analysis for treatment of male infertility. Transl Androl Urol.

[29] Suh RS, Zhu X, Phadke N, Ohl DA, Takayama S, Smith GD (2006). IVF within microfluidic channels requires lower total numbers and lower concentrations of sperm. Hum Reprod.

[30] Zeringue HC, Beebe DJ (2004). Microfluidic removal of cumulus cells from Mammalian zygotes. Methods Mol Biol.

[31] Lu Z, Zhang X, Leung C, Esfandiari N, Casper RF, Sun Y (2011). Robotic ICSI (intracytoplasmic sperm injection). IEEE Trans Biomed Eng.

[32] Mor A, Zhang M, Esencan E (2020). A step towards the automation of intracytoplasmic sperm injection: real time confirmation of mouse and human oocyte penetration and viability by electrical resistance measurement. Fertil Steril.

[33] Yang Q, Zhu L, Jin L (2020). Human follicle in vitro culture including activation, growth, and maturation: a review of research progress. Front Endocrinol (Lausanne).

[34] McLaughlin M, Albertini DF, Wallace WH, Anderson RA, Telfer EE (2018). Metaphase II oocytes from human unilaminar follicles grown in a multi-step culture system. Mol Hum Reprod.

[35] Laronda MM, Rutz AL, Xiao S (2017). A bioprosthetic ovary created using 3D printed microporous scaffolds restores ovarian function in sterilized mice. Nat Commun.

[36] Salama M, Woodruff TK (2019). From bench to bedside: current developments and future possibilities of artificial human ovary to restore fertility. Acta Obstet Gynecol Scand.

[37] Xiao S, Coppeta JR, Rogers HB (2017). A microfluidic culture model of the human reproductive tract and 28-day menstrual cycle. Nat Commun.

[38] Quinn MM, Cakmak H, Letourneau JM, Cedars MI, Rosen MP (2017). Response to ovarian stimulation is not impacted by a breast cancer diagnosis. Hum Reprod.

[39] Mai Q, Hu X, Yang G, Luo Y, Huang K, Yuan Y, Zhou C (2017). Effect of letrozole on moderate and severe early-onset ovarian hyperstimulation syndrome in high-risk women: a prospective randomized trial. Am J Obstet Gynecol.

[40] Goldrat O, Gervy C, Englert Y, Delbaere A, Demeestere I (2015). Progesterone levels in letrozole associated controlled ovarian stimulation for fertility preservation in breast cancer patients. Hum Reprod.

[41] Pereira N, Hancock K, Cordeiro CN, Lekovich JP, Schattman GL, Rosenwaks Z (2016). Comparison of ovarian stimulation response in patients with breast cancer undergoing ovarian stimulation with letrozole and gonadotropins to patients undergoing ovarian stimulation with gonadotropins alone for elective cryopreservation of oocytes†. Gynecol Endocrinol.

[42] Bosdou JK, Venetis CA, Kolibianakis EM, Toulis KA, Goulis DG, Zepiridis L, Tarlatzis BC (2012). The use of androgens or androgen-modulating agents in poor responders undergoing in vitro fertilization: a systematic review and meta-analysis. Hum Reprod Update.

[43] Gleicher N, Weghofer A, Barad DH (2010). Dehydroepiandrosterone (DHEA) reduces embryo aneuploidy: direct evidence from preimplantation genetic screening (PGS). Reprod Biol Endocrinol.

[44] Gleicher N, Ryan E, Weghofer A, Blanco-Mejia S, Barad DH (2009). Miscarriage rates after dehydroepiandrosterone (DHEA) supplementation in women with diminished ovarian reserve: a case control study. Reprod Biol Endocrinol.

[45] Jinek M, Chylinski K, Fonfara I, Hauer M, Doudna JA, Charpentier E (2012). A programmable dual-RNA-guided DNA endonuclease in adaptive bacterial immunity. Science.

[46] Kang E, Wu J, Gutierrez NM (2016). Mitochondrial replacement in human oocytes carrying pathogenic mitochondrial DNA mutations. Nature.

